# Reducing Dietary Protein Content by Increasing Carbohydrates Is More Beneficial to the Growth, Antioxidative Capacity, Ion Transport, and Ammonia Excretion of Nile Tilapia (*Oreochromis niloticus*) under Long-Term Alkalinity Stress

**DOI:** 10.1155/2023/9775823

**Published:** 2023-11-16

**Authors:** Wei Liu, Chang Xu, Zhao Li, Liqiao Chen, Xiaodan Wang, Erchao Li

**Affiliations:** ^1^Key Laboratory of Tropical Hydrobiology and Biotechnology of Hainan Province, Hainan Aquaculture Breeding Engineering Research Center, School of Marine Biology and Aquaculture, Hainan University, Haikou 570228, China; ^2^School of Life Sciences, East China Normal University, Shanghai 200241, China

## Abstract

Alkalinity stress is the main stress experienced by aquatic animals in saline–alkali water, which hinders the aquaculture development and the utilization of water resources. The two-factor (2 × 3) test was adopted to study the influence of dietary protein to carbohydrate ratios on the energy metabolism of Nile tilapia (*Oreochromis niloticus*) under different alkalinity stress levels. Three diets with different protein-carbohydrate ratios (P27/C35, P35/C25, and P42/C15) were fed to fish cultured in freshwater (FW, 1.3 mmol/L carbonate alkalinity) or alkaline water (AW, 35.7 mmol/L carbonate alkalinity) for 50 days. Ambient alkalinity decreased tilapia growth performance. Although ambient alkalinity caused oxidative stress and enhanced ion transport and ammonia metabolism in tilapia, tilapia fed the P27/C35 diet showed better adaptability than fish fed the other two diets in alkaline water. Further metabolomic analysis showed that tilapia upregulated all the pathways enriched in this study to cope with alkalinity stress. Under alkalinity stress, tilapia fed the P27/C35 diet exhibited enhanced pyruvate metabolism and purine metabolism compared with tilapia fed the P42/C15 diet. This study indicated that ambient alkalinity could significantly decrease growth performance and cause oxidative stress and osmotic regulation. However, reducing dietary protein content by increasing carbohydrates could weaken stress and improve growth performance, ion transport, and ammonia metabolism in tilapia under long-term hyperalkaline exposure.

## 1. Introduction

Aquaculture makes an enormous contribution to global food security and provides essential nutritional support to humans [1]. However, limited aquaculture water has seriously restricted the development of aquaculture [2]. Therefore, the development of new aquaculture styles using different water resources is of great significance to maintain the sustainable development of aquaculture [3]. Saline–alkali water, formed by long-term evaporation, drought, or little rain, is widely distributed in more than 100 countries, and the area of saline–alkali land accounts for one-third of the world's total land area [4–6]. Thus, the development of saline–alkaline water would be an effective way to enhance aquaculture development. Meanwhile, aquatic animals will face worse salinization and alkalinization problems in water under global climate change [7]. Therefore, it is essential to understand the adaptation mechanism of aquatic animals to saline–alkali conditions and develop strategies to improve saline–alkali adaptation.

Alkalinity stress is considered the main stress in saline–alkali water habitats and can suppress the growth, reproduction, development, survival, and even intestinal microflora of aquatic animals [8, 9]. Alkalinity stress increases the blood HCO_3_^−^ concentration and disrupts the balance of NH_3_ and NH_4_^+^ due to a lack of H^+^, which finally results in ammonia poisoning [10]. Alkalosis and ammonia poisoning can cause abnormal amino acid metabolism, which is a main reason for the poor protein utilization and suppressed growth of aquatic animals in saline–alkaline water [8]. Protein is a vital nutrient for the growth of fish [11]. However, protein metabolism also increases the burden of ammonia accumulation [8], which may aggravate ammonia poisoning under alkalinity stress. It has been confirmed in some studies that high dietary protein could aggravate the stress of alkalinity for aquatic animals. The results showed that higher dietary protein contents resulted in worse impairment of intestinal microflora homeostasis, immune function, and growth performance under alkalinity stress [8]. The metabolic mechanism of aquatic animals under carbonate alkalinity stress is not clear.

Carbohydrates are another important nutrient in aquatic animal feed in addition to protein, especially during adaptation to environmental stress [6, 12, 13]. It could satisfy an increasingly rapid energy demand of animals coping with stress [14, 15]. Moreover, it could also exert protein-sparing effects, which would have good economic benefits [16]. Low dietary protein levels mean that less ammonia is produced by protein catabolism, which might also help alleviate the ammonia poisoning caused by alkalinity stress [17]. However, the influence of alkalinity stress on carbohydrate and protein metabolism and the appropriate dietary protein–carbohydrate ratio to help aquatic animals better cope with alkalinity stress are still unclear.

Tilapia are the main popular aquaculture species farmed in 127 countries because of their abundant nutritional value and economic advantages [18, 19]. Nile tilapia (*Oreochromis niloticus*) is the primary farmed tilapia species on account of its high nutritional value and tolerance against stress [20]. It also has good utilization of various carbohydrates [21]. Moreover, domesticated Nile tilapia can tolerate a limited range of alkaline and saline stresses [22]. Therefore, Nile tilapia is a good model organism for studying the influences of alkalinity stress on metabolism and developing nutritional strategies to improve alkalinity tolerance [23, 24]. In this study, Nile tilapia was subjected to chronic alkalinity stress to research the influences of different protein-carbohydrate ratios on the growth performance, antioxidant capacity, ammonia metabolism, ion transport, and metabolomics. The results will help to expound the potential mechanism of coping with alkalinity stress in Nile tilapia and provide estimable information for nutrient metabolism and breeding excellent varieties with better performance under alkalinity stress. Thus, it can help to develop saline–alkali water for aquaculture.

## 2. Materials and Methods

### 2.1. Experimental Animals and Experimental Design

Three isoenergies (16.5 KJ/g) and isolipidic (60 g/kg) diets were formulated with three protein–carbohydrate ratios (P27/C35, 27% crude protein with 35% carbohydrate; P35/C25, 35% crude protein with 25% carbohydrate; and P42/C15, 42% crude protein with 15% carbohydrate). The corn starch, fish meal, and soybean meal were used as dietary carbohydrate and protein sources. The detailed composition of the fish feed is shown in Table 1. All raw materials were pulverized into powder and then sifted through a 60-mesh sieve and thoroughly mixed before being extruded into 2-mm diameter pellets in a double helix extruder (model: CD4-1TS). The air-dried diets at room temperature were stored at −20°C until use. These were two types of water (freshwater, FW, carbonate alkalinity = 1.3 mmol/L and alkaline water, AW, carbonate alkalinity = 35.7 mmol/L). Freshwater was prepared using tap-water aerated for 1 day before use. Carbonate–alkalinity water was prepared using NaHCO_3_ and dechlorinated tap water and aerated for 24 hr before use.

Juvenile Nile tilapia was acquired from a tilapia company in Wenchang (Hainan, China). Before the test, tilapia was acclimatized for 2 weeks in two tanks (500 L). In the last 5 days of this period, a tank was randomly selected and injected with a set concentration of alkaline water (alkalinity increased approximately 7 mmol/L per day) by changing the water. After acclimation, 225 healthy fish (0.45 ± 0.02 g) living in freshwater were randomly divided into three groups: LF, fish fed the P27/C35 diet in freshwater; MF, fish fed the P35/C25 diet in freshwater; and HF, fish fed the P42/C15 diet in freshwater, and 225 healthy fish (0.45 ± 0.02 g) living in alkaline water were randomly divided into three groups: LA, fish fed the P27/C35 diet in alkaline water; MA, fish fed the P35/C25 diet in alkaline water; and HA, fish fed the P42/C15 diet in alkaline water. The 18 tanks (60 cm × 30 cm × 35 cm, 25 fish per tank) were randomly assigned to six groups with three replicates in each group. The trial lasted for 50 days. During the test, tilapia was fed twice a day at 08 : 00 and 16 : 00 until obvious repletion, and daily food consumption was accurately recorded per tank. The daily exchange of water was 70% of the total volume, and the water quality parameters were kept at 28.2–30.5°C and dissolved oxygen >7.0 mg/L.

### 2.2. Sample Collection

Before sampling, fish were starved for 1 day and then anesthetized with MS-222 (30–60 mg/L) from each tank. The length, weight, and number of all tilapia were recorded to calculate the growth performance. Three fish from each group were randomly selected, and tail vein blood was drawn using a syringe containing heparin sodium. The blood sample was placed at 4°C overnight and centrifuged in a refrigerated centrifuge (3–18 KS, Sigma, Osterode am Harz, Germany) for 5 min (4°C, 2,500 g). The hemolymph supernatant plasma was stored at −80°C for further analysis. The liver and gill of another 12 fish per tank were taken and placed into 1.5-mL enzyme-free tubes, rapidly frozen in liquid nitrogen, and then kept at −80°C for future analysis, and the liver was numbered and weighed to compute the hepatosomatic index (HSI) before freezing.

### 2.3. Biochemical Analysis

The kits utilized for measuring the concentration of plasma ammonia (A086-1-1) were acquired from Nanjing Jiancheng Bioengineering Institute. Six fish from each group (*n* = 6) were collected and homogenized (1 : 9, w/v) in cold 0.86% NaCl. The homogenates were then centrifuged at 3,500 rpm at 4°C for 10 min in a centrifuge tube. Liver homogenates were made to determine malonaldehyde (MDA) contents and catalase (CAT), superoxide dismutase (SOD), and glutathione peroxidase (GSH-PX) activities. All experimental procedures were performed according to the instructions provided by the kit manufacturer (Jiancheng, Ltd., Nanjing, China).

### 2.4. Quantitative Real-Time PCR

Six fish from each group (*n* = 6) were randomly selected for RNA extraction. Total RNA from the livers and gills was extracted by Trizol reagent (GLPBIO, USA) according to a previously described method [26]. The total RNA concentration was adjusted to 5,00 ng/*μ*L using a Nanodrop2000. Reverse transcription into cDNA was performed using a reverse transcription kit (Biosharp, China). The primers designed by Primer 5 software for qPCR are shown in Table 2, and *β-actin* was used as the internal reference gene. An RT-PCR kit was used for fluorescence quantification. The relative expression (fold changes) of the target gene was estimated by using the 2^−*ΔΔ*Ct^ method [27].

### 2.5. Metabolomics Analysis

#### 2.5.1. Metabolite Extraction

Six fish from each group (LF, HF, LA, and HA) (*n* = 6) were randomly selected for metabolite extraction. Twenty-five milligrams of fish liver were weighed and placed in an EP tube with 500 *μ*L of extracting solution (methanol : acetonitrile : water = 2 : 2 : 1, an internal standard mixture labeled with isotopes). Then, the samples were homogenized and sonicated. After that, the samples were incubated and centrifuged. The resulting supernatant was transferred to a fresh glass bottle for analysis. Quality control samples were prepared by mixing an equivalent quantity of the liquid supernatant from all samples.

#### 2.5.2. LC‒MS/MS Analysis

Six fish from each group (LF, HF, LA, and HA) (*n* = 6) were randomly selected for LC‒MS/MS analyses. The UHPLC system (Vanquish, Thermo Fisher Scientific) was used for LC–MS/MS analysis. The Orbitrap Exploris 120 mass spectrometer was used for its ability to acquire MS/MS spectra in information-dependent acquisition mode in the control of the acquisition software (Xcalibur, Thermo). The ESI source conditions were set as follows: sheath gas flow rate of 50 Arb, aux gas flow rate of 15 Arb, capillary temperature of 320°C, full MS resolution of 60,000, MS/MS resolution of 15,000 collision energy of 10/30/60 in NCE mode, and spray voltage of 3.8 kV (positive) or −3.4 kV (negative).

#### 2.5.3. Data Preprocessing and Annotation

ProteoWizard was used to convert raw data into mzXML format and processed with the in-house program that was developed using R and based on XCMS for peak detection, extraction, alignment, and integration. The in-house MS2 database (BiotreeDB) was used for metabolite annotation. The cutoff for annotation was set at 0.3.

### 2.6. Statistical Analysis

SPSS Statistics 26 (IBM, Armonk, NY, USA) was used for all statistical analyses. The data are expressed as the mean ± standard error of the mean (SEM). All data met the normal distribution and variance homogeneity test. Two-way ANOVA of variance was used to analyze the significance of the main influence of alkalinity, dietary protein to carbohydrate ratios, and their interaction. Then, one-way ANOVA followed by Duncan's multiple comparison test was adopted to analyze data from the same alkalinity level groups with the different dietary protein–carbohydrate ratios. The independent-samples *t*-test was used to analyze data from the same protein–carbohydrate ratio groups within the different alkalinity levels. *P* < 0.05 was regarded as statistically significant. Correlation network heatmap analysis was performed using https://www.omicshare.com.

## 3. Results

### 3.1. Growth and Physiological Parameters

The growth performance of the fish in each group is shown in Table 3. There was no significant difference in SR among all groups (*P* > 0.05). The WG, SGR, and FCR were significantly affected by alkalinity, dietary protein to carbohydrate ratios, and their interaction (*P* < 0.05). The HSI was markedly influenced by dietary protein to carbohydrate ratios and the interaction between alkalinity and dietary protein to carbohydrate ratios (*P* < 0.05). The CF was markedly influenced by alkalinity and the interaction between alkalinity and dietary protein to carbohydrate ratios (*P* < 0.05). For the same diets, the tilapia in freshwater had markedly higher WG and SGR and lower FCR than those in alkaline water (*P* < 0.05), but significantly lower CF values were found only in tilapia fed the P42/C15 diet in alkaline water than in freshwater. In freshwater, tilapia fed the P42/C15 diet had a markedly lower SGR and higher CF than tilapia fed the other two diets (*P* < 0.05). Specifically, under alkaline water, the values of WG, SGR, HSI, and CF in tilapia fed the P42/C15 diet were lower than those in tilapia fed the other two diets (*P* < 0.05), but the values of FCR in tilapia fed the P42/C15 diet were highest (*P* < 0.05).

### 3.2. Antioxidative Parameters in Livers

**Figure 1:**
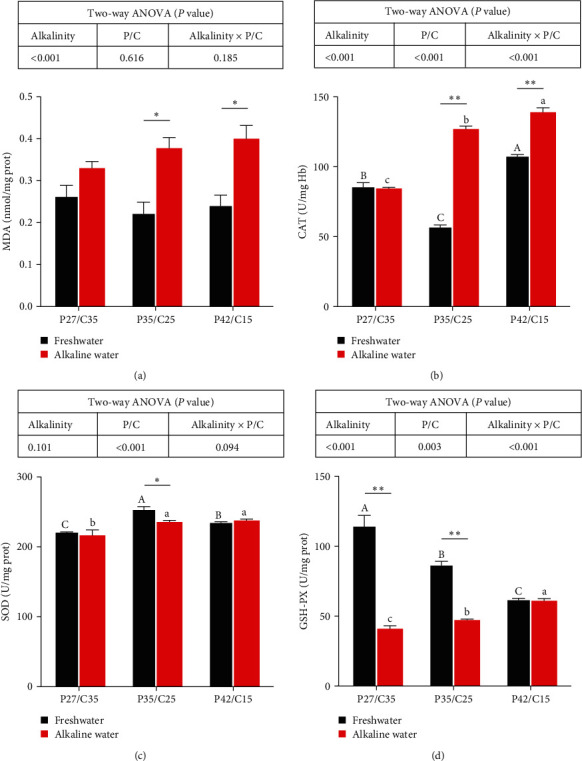
Activities of MDA (a), CAT (b), SOD (c), and GSH-PX (d) in the liver of *O. niloticus* fed different dietary protein to carbohydrate ratios in freshwater or alkaline water for 50 days. All data are represented as the mean ± SEM (*n* = 6). Different letters in the same histogram show significant differences (*P* < 0.05).  ^*∗*^Indicates a significant difference between different alkalinity levels within the same diet protein–carbohydrate ratios (*P* < 0.05).  ^*∗∗*^Indicates a significant difference between different alkalinity levels within the same diet protein–carbohydrate ratios (*P* < 0.01).

There was a significant main influence of alkalinity, dietary protein to carbohydrate ratios, and their interaction on the CAT and GSH-PX activities (*P* < 0.05). The MDA content was markedly affected by alkalinity (*P* < 0.05). The diet protein–carbohydrate ratios affected SOD activities (*P* < 0.05). Dramatically higher MDA (Figure 1(a)) contents were found in tilapia in alkaline water than in freshwater except for tilapia fed the P27/C35 diet (*P* < 0.05). Tilapia fed P35/C25 and P42/C15 diets in alkaline water showed markedly higher CAT activities (Figure 1(b)) than tilapia in freshwater (*P* < 0.05). Tilapia fed P27/C35 and P35/C25 diets in alkaline water had markedly lower GSH-PX activities (Figure 1(d)) than those in freshwater (*P* < 0.05). However, tilapia fed only the P35/C25 diet in freshwater had markedly higher SOD activities (Figure 1(c)) than those in alkaline water (*P* < 0.05). Under freshwater, the highest CAT activities and the lowest GSH-PX activities were observed in tilapia fed the P42/C15 diet (*P* < 0.05), and the tilapia fed the P35/C25 diet had the highest SOD activities (*P* < 0.05). However, the highest CAT, SOD, and GSH-PX activities were all found in tilapia fed the P42/C15 diet under alkaline water (*P* < 0.05).

### 3.3. Ion Transport in Gills

**Figure 2:**
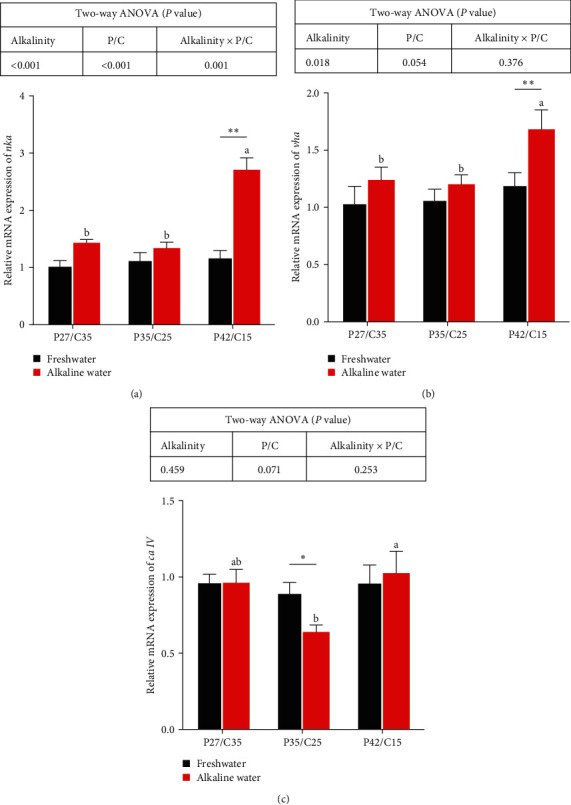
Expression of genes related to Na^+^−K^+^−ATPase (a), V-H^+^−ATPase (b), and carbonic anhydrase IV (c) in gills of *O. niloticus* fed different dietary protein to carbohydrate ratios in freshwater or alkaline water for 50 days. Data are expressed as the mean ± SEM (standard error of the mean) (*n* = 6). Different letters in the same histogram show significant differences (*P* < 0.05).  ^*∗*^Indicates a significant difference between different alkalinity levels within the same diet protein–carbohydrate ratios (*P* < 0.05).  ^*∗∗*^Indicates a significant difference between different alkalinity levels within the same diet protein–carbohydrate ratios (*P* < 0.01). *nka*, Na^+^−K^+^−ATPase; *vha*, V-H^+^−ATPase; and *ca IV*, carbonic anhydrase IV.

The mRNA levels of Na^+^−K^+^−ATPase (*nka*) were influenced by alkalinity, dietary protein to carbohydrate ratios, and their interaction (*P* < 0.05). The mRNA levels of V-H^+^−ATPase (*vha*) were affected by alkalinity (*P* < 0.05). The expression of *nka* (Figure 2(a)) and *vha* (Figure 2(b)) was prominently higher in tilapia fed only the P42/C25 diet in alkaline water than in those fed freshwater (*P* < 0.05). Tilapia fed the P35/C25 diet in alkaline water had markedly lower expression of carbonic anhydrase IV (*ca IV*) (Figure 2(c)) than those in freshwater (*P* < 0.05). No marked difference was shown in the mRNA levels of *nka*, *vha*, and *ca IV* among all freshwater groups (*P* > 0.05). Specifically, the highest gene expression of *nka*, *vha*, and *ca IV* was found in tilapia fed the P42/C25 diet among all alkaline water groups (*P* < 0.05).

### 3.4. Ammonia Metabolism in Livers

**Figure 3:**
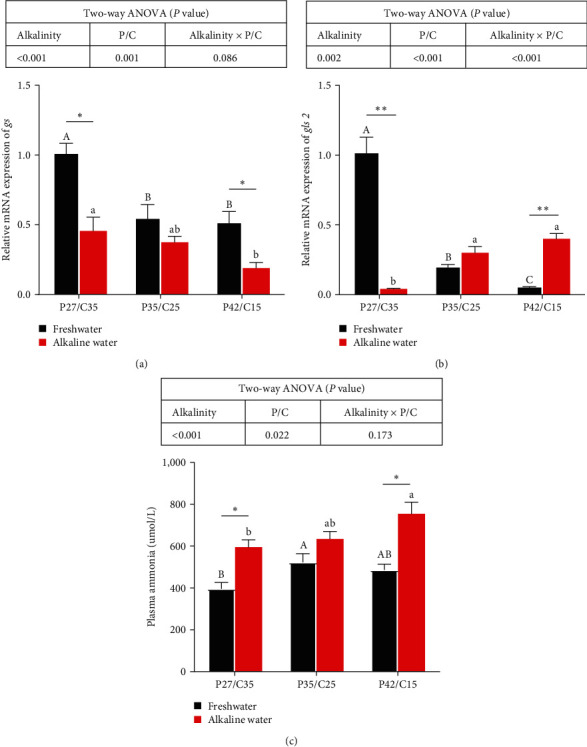
Expression of genes related to glutamine synthetase (a) and glutaminase 2 (b) in the liver and plasma ammonia (c) of *O. niloticus* fed different dietary protein to carbohydrate ratios in freshwater or alkaline water for 50 days. Data are expressed as the mean ± SEM (standard error of the mean) (*n* = 3). Different letters in the same histogram show significant differences (*P* < 0.05).  ^*∗*^Indicates a significant difference between different alkalinity levels within the same diet protein–carbohydrate ratios (*P* < 0.05).  ^*∗∗*^Indicates a significant difference between different alkalinity levels within the same diet protein–carbohydrate ratios (*P* < 0.01). *gs*, glutamine synthetase and *gls 2*, glutaminase 2.

The gene expression of glutamine synthetase (*gs*) was significantly affected by alkalinity and dietary protein to carbohydrate ratios (*P* < 0.05). The gene expression of glutaminase 2 (*gls 2*) was markedly affected by alkalinity, dietary protein to carbohydrate ratios, and their interaction (*P* < 0.05). Meanwhile, the plasma ammonia content of tilapia was prominently affected by alkalinity (*P* < 0.05). Dramatically higher gene expression of *gs* (Figure 3(a)) was found in tilapia in freshwater than in alkaline water except for tilapia fed the P35/C25 diet (*P* < 0.05). Specifically, higher gene expression of *gls 2* (Figure 3(b)) was found in tilapia fed the P27/C35 diet in freshwater than in alkaline water (*P* < 0.05), while tilapia fed the P42/C15 diet in freshwater had lower gene expression of *gls 2* than those in alkaline water (*P* < 0.05). In addition, significantly higher plasma ammonia content (Figure 3(c)) was found in tilapia in alkaline water than in freshwater except for tilapia fed the P35/C25 diet (*P* < 0.05). Under freshwater, tilapia fed the P27/C35 diet had the highest expression of *gs* and *gls 2* and the lowest plasma ammonia content compared with the other two groups (*P* < 0.05). However, the lowest expression of *gs* and the highest *gls 2* gene expression level and plasma ammonia content were found in tilapia fed the P27/C35 diet among all alkaline water groups (*P* < 0.05).

### 3.5. Association between Antioxidant Capacity, Ion Transport, and Ammonia Metabolism

**Figure 4:**
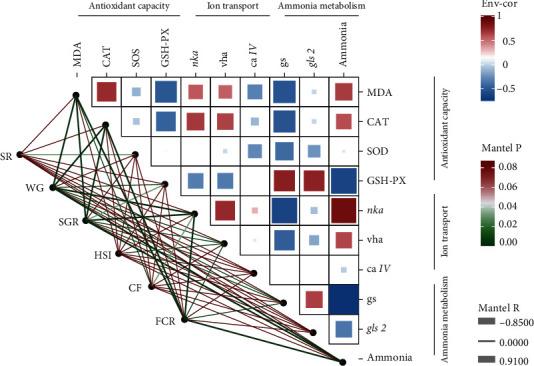
Correlations among growth performance, biochemical analysis, and quantitative RT-PCR. The edge width of lines refers to Mantel's *r* for the statistics of corresponding distance correlations, and the color of lines represents the statistical significance.

As shown in Figure 4, the growth performance of tilapia was closely related to antioxidant capacity, ion transport, and ammonia metabolism. The WG and SGR were significantly negatively correlated with the MDA content, the CAT activity, the mRNA levels of *nka* and *vha* and plasma ammonia content (*P* < 0.05), but significantly positively correlated with the mRNA levels of *gs* (*P* < 0.05). In the meantime, the FCR was significantly positively correlated with the MDA content, the CAT activity, the mRNA levels of *nka* and *vha*, and plasma ammonia content (*P* < 0.05), but significantly negatively correlated with the mRNA levels of *gs* (*P* < 0.05). Besides, there were significant relationships among the other parameters.

### 3.6. Liver Metabolomics

**Figure 5:**
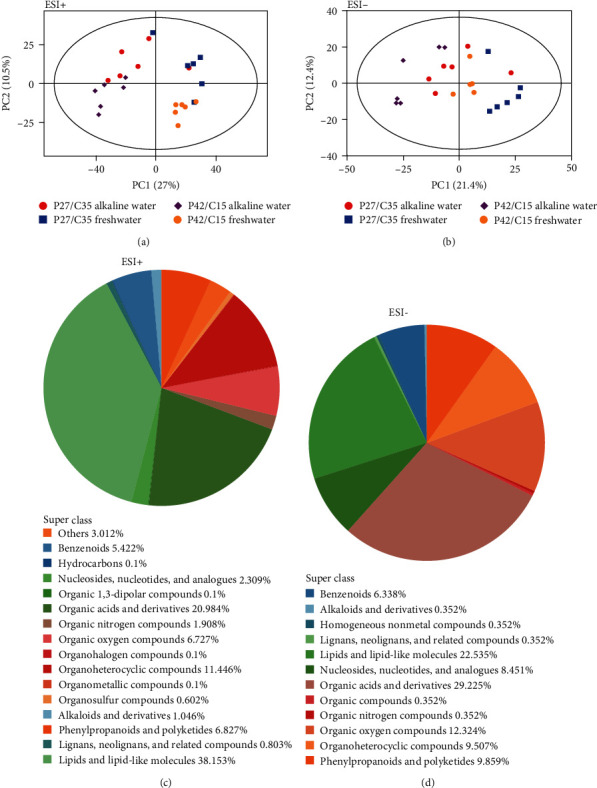
Principal component analysis of different metabolites of Nile tilapia fed different protein–carbohydrate ratios in freshwater or alkaline water for 50 days (*n* = 6). (a) The quantitative proportion of chemical classification of metabolites in positive ion mode. (b) The quantitative proportion of chemical classification of metabolites in negative ion mode. (c) PCA score plot of positive ion mode. (d) PCA score plot of negative ion mode.

Positive ion mode (POS) and negative ion mode (NEG) were used to detect tilapia in LF group, the LA group, and the HA group by mass spectrometry. A total of 13,556 and 12,044 metabolites were found in POS and NEG, respectively. The PCA score plot (Figures 5(a) and 5(b)) showed that all live samples were within 95% confidence intervals. In POS (Figure 5(c)), metabolites were divided into 16 classes according to chemical classification, such as lipids and lipid-like molecules (38.153%), organic acids and derivatives (20.984%), and organoheterocyclic compounds (11.446%). In NEG (Figure 5(d)), metabolites were divided into 12 classes, including organic acids and derivatives (29.225%), lipids and lipid-like molecules (22.535%), and organoheterocyclic compounds (12.324%).

**Figure 6:**
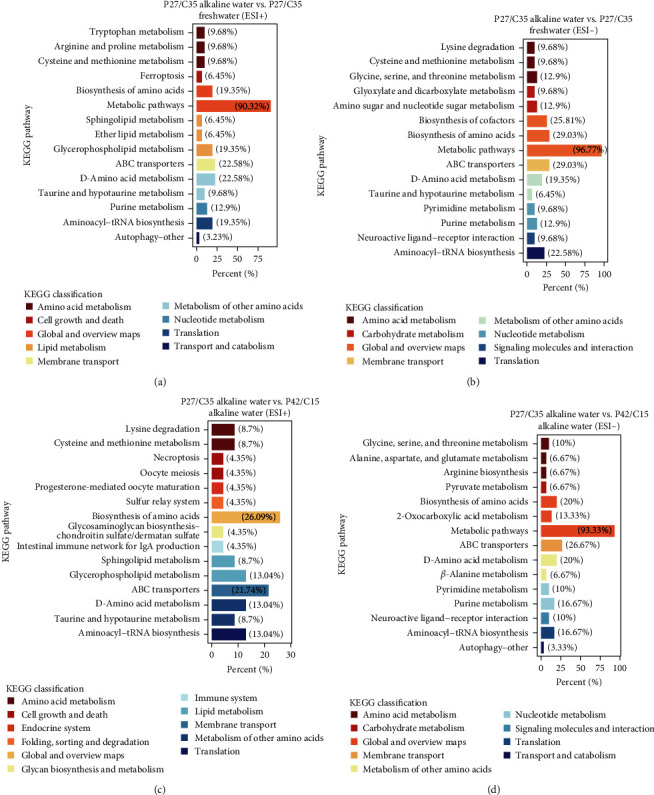
Screening and enrichment analysis of different metabolites among the three groups (*n* = 6). (a) KEGG classification and KEGG pathway analysis of different metabolites between P27/C35 alkaline water and P27/C35 freshwater in positive ion mode. (b) KEGG classification and KEGG pathway analysis of different metabolites between P27/C35 alkaline water and P27/C35 freshwater in negative ion mode. (c) KEGG classification and KEGG pathway analysis of different metabolites between P27/C35 alkaline water and P42/C15 alkaline water in positive ion mode. (d) KEGG classification and KEGG pathway analysis of different metabolites between P27/C35 alkaline water and P42/C15 alkaline water in negative ion mode.

**Figure 7:**
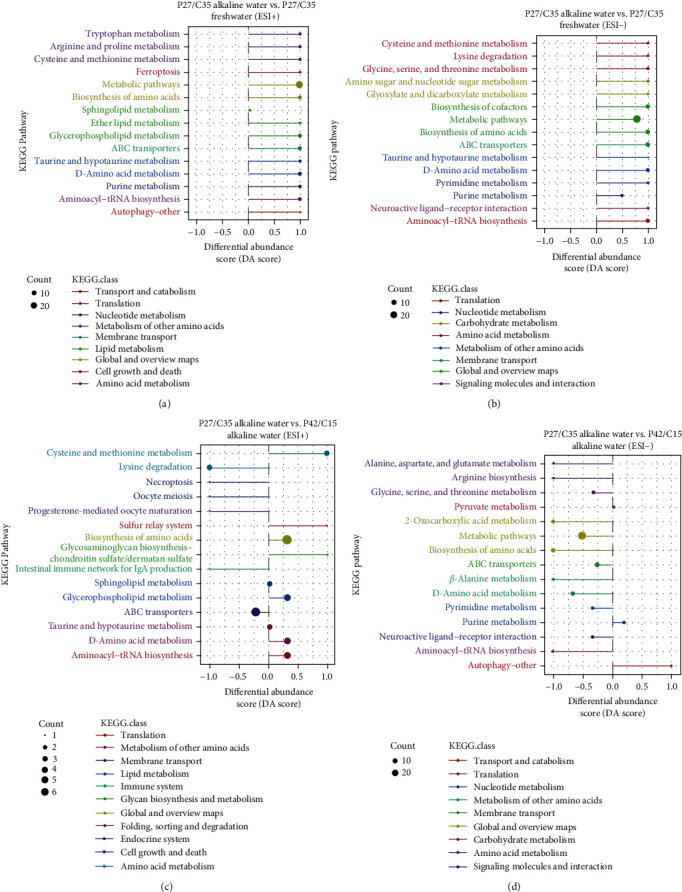
Expression trend of different metabolites among the three groups (*n* = 6). (a) Differential abundance scores for all enriched metabolic pathways between P27/C35 alkaline water and P27/C35 freshwater in positive ion mode. (b) Differential abundance scores for all enriched metabolic pathways between P27/C35 alkaline water and P27/C35 freshwater in negative ion mode. (c) Differential abundance scores for all enriched metabolic pathways between P27/C35 alkaline water and P42/C15 alkaline water in positive ion mode. (d) Differential abundance scores for all enriched metabolic pathways between P27/C35 alkaline water and P42/C15 alkaline water in negative ion mode.

The metabolites of each group were screened for different metabolites (DMs). Then, according to KEGG enrichment analysis, DMs (LA vs. LF) were markedly enriched in metabolic pathways (90.32%), ABC transporters (22.58%), and D-amino acid metabolism (22.58%) in POS (Figure 6(a)), and DMs (LA vs. HA) were significantly enriched in metabolic pathways (96.77%), biosynthesis of amino acids (29.03%), and ABC transporters (29.03%) in NEG (Figure 6(b)). For LA vs. HA, DMs were observed to be significantly enriched in biosynthesis of amino acids (26.09%), ABC transporters (21.74%), and D-amino acid metabolism (13.04%) in POS (Figure 6(c)), and DMs were observed to be significantly enriched in metabolic pathways (93.33%), ABC transporters (26.67%), and D-amino acid metabolism (20%) in NEG (Figure 6(d)). Furthermore, the overall changes in metabolites within the enriched metabolic pathways were analyzed. The metabolite expression of metabolic pathways in the LA group all showed an upregulated trend compared with that in the LF group regardless of POS (Figure 7(a)) or NEG (Figure 7(b)). At the same time, the LA group upregulated cysteine and methionine metabolism and downregulated lysine degradation, necroptosis, and oocyte meiosis pathways compared with the HA group in POS (Figure 7(c)). Additionally, compared with the HA group in the NEG (Figure 7(d)), the LA group showed downregulation in all metabolic pathways except for pyruvate metabolism, purine metabolism, and autophagy.

## 4. Discussion

Environmental factors can affect the growth performance of aquatic animals by changing nutrient metabolism and absorption [28]. Previous studies have found that alkalinity stress can hinder normal ammonia excretion, cause abnormal amino acid metabolism, and eventually inhibit growth [8]. Additionally, the growth performance of *Chalcalburnus chalcoides aralensis* under carbonate alkalinity (40.78 mmol/L) for 30 days was also significantly inhibited [29]. In this study, tilapia in alkaline water showed lower WG and SGR and higher FCR than those in freshwater. This is because aquatic animals need to expend a significant amount of energy to cope with alkalinity stress, thereby reducing the energy available for growth [30]. However, under alkalinity stress, tilapia fed the high-carbohydrate diet had the higher WG and SGR and lower FCR than those fed the high-protein diet in this study, which meant that the suppressed growth of tilapia was alleviated by the increase in dietary carbohydrates under alkalinity stress. This may be because tilapia fed with high-carbohydrate diet could better cope with osmotic regulation caused by alkalinity stress. Carbohydrates are the direct energy supply to aquatic animals under salinity stress [14, 31, 32]. The correlation analysis of this study also showed that WG and SGR were significantly negatively correlated with ion transport, while FCR was significantly positively correlated with ion transport. In addition, the ability to avoid ammonia poisoning is a determinant of carbonate alkalinity stress tolerance [29, 33]. Meanwhile, this study found that the WG, SGR, and FCR were negatively correlated with plasma ammonia. Therefore, tilapia fed the low-protein diet had the lower plasma ammonia contents under alkalinity stress in this study, which may be another reason for their better growth performance.

The toxic effect of alkalinity is the synergistic result of pH and CO_3_^2−^ [5, 6]. The gill is often used as a biomarker of aquatic toxicology because it is directly exposed to the habitat environment [34]. Carbonate alkalinity stress will impair the structure of gills and break the ion balance of CO_3_^2−^ and HCO_3_^−^, which would then unbalance the acid–base balance [6, 10, 29]. Studies have proven that *nka* is an important ion pump for osmotic regulation of fish that the excretion of H^+^ via *vha* promotes branchial ammonia excretion by capturing the excreted NH_3_ as NH_4_^+^ [35–37]. In this study, tilapia in alkaline water had higher gene expression of *nka* and *vha* than that in freshwater, which indicated that tilapia needed ion regulation to maintain their osmotic balance and acid–base balance under alkalinity stress. In addition, carbonic anhydrase can maintain acid–base balance in fish gills [38], and CO_2_ exhaled by fish is transformed into HCO_3_^−^ and H^+^ by carbonic anhydrase in gills, which promotes ammonia excretion [37, 39]. Our study showed that the *ca IV* mRNA expression of fish in alkaline water was lower than that in freshwater, which may be due to the inhibition of carbonic anhydrase mRNA expression caused by ammonia metabolic burden [37]. After alkalinity disturbs the ion balance and acid–base regulation in the body, the lack of H^+^ caused by acid–base imbalance will also lead to the obstruction of ammonia emission and accumulation of plasma ammonia in vivo [5, 29, 40]. In summary, alkaline water often negatively affects the ammonia metabolic burden [3]. Therefore, the plasma ammonia concentration of fish in alkaline water was greater than that of fish in freshwater in this study. Glutamine metabolism is an important way for alkali-tolerant fish to adapt to alkaline environments because ammonia and glutamate can be catalyzed into nontoxic glutamine by *gs*, which excrete ammonia efficiently [41]. Besides, the product of protein catabolism is ammonia, and fish fed more protein usually have higher plasma ammonia content, which leads to fish fed high-protein diets having higher gene expression of *gs* to excrete more ammonia from their bodies [42–44]. Specifically, high plasma ammonia contents could suppress the growth of fish [45], which may be why tilapia fed the high-protein diet in alkaline water had the worst growth performance in this study. The results showed that a low-protein diet can reduce the burden of ammonia metabolism in tilapia under alkalinity stress. In summary, to cope with alkalinity stress, tilapia can activate the osmotic pressure, ammonia metabolism, and acid–base balance adjustment mechanisms to maintain the physiological balance. Specifically, this physiological process involves the interaction of numerous genes and metabolites and consumes more energy. Carbohydrate catabolism not only does not cause ammonia metabolic burden but can also directly supply energy to help the body cope with stress [15, 17, 31]. Therefore, reducing dietary protein content by increasing carbohydrates was more beneficial to ammonia metabolism in tilapia under alkalinity stress.

Both carbonate alkalinity stress and ammonia poisoning can entice the formation of reactive oxygen species (ROS) [24, 46], thus influencing the antioxidant defense system and growth of fish. As one of the peroxidation products in the process of substance metabolism, the increase in MDA content is one of the indicators of oxidative damage [47]. Alkalinity can significantly increase MDA content [8]. The increase in MDA content indicates the occurrence of lipid peroxidation, which generates a large amount of ROS [48]. However, the antioxidant enzyme system (CAT, SOD, and GSH-PX) has the ability to clear ROS in vivo [49]. This study showed that long-term alkalinity stress led to an increase in MDA content and a decrease in SOD and GSH-PX activities, indicating decreased antioxidant capacity in the liver. Studies have shown that alkalinity can damage the antioxidant system of *Eriocheir sinensis* and *Cyprinus carpio Songpu* [8, 29]. Specifically, the lowest CAT, SOD, and GSH-PX activities were observed in tilapia fed the low-protein diet under alkalinity stress in this study. The reason may be that the low-protein diet could reduce the ammonia metabolic burden of fish in saline–alkali water [34, 35], thus reducing oxidative stress. Similar to our findings, studies in *Cyprinus carpio Songpu* have shown that a decrease in dietary protein levels could reduce free radical production and oxidative stress caused by alkalinity stress [8]. In addition, the antioxidant defense system seems to be closely related to energy metabolism [32, 50]. In this study, the low-protein diet group had more carbohydrates, and carbohydrate catabolism can provide much energy for the body's response to oxidative stress. Therefore, reducing dietary protein content by increasing carbohydrates can not only maintain the stability of energy metabolism but also be more conducive to the stability of the antioxidant defense system in tilapia under long-term alkalinity stress.

Aquatic animals can tolerate or compensate for harsh environments by changing their metabolism [28]. Metabolomics is thought to be a promising tool to monitor stress responses when exposed to environmental stress [51]. In alkaline water, the toxicity of HCO_3_^−^ poses serious challenges to the growth of fish [10]. The metabolism of tilapia in alkaline water was activated to maintain homeostasis in this study. Trioxypurines produced by purine metabolism and arginine metabolism are the main sources of urea synthesis in tilapia [52]. The results showed that the purine metabolism and arginine metabolism of tilapia in alkaline water rose significantly, which meant that tilapia could relieve the burden of ammonia metabolism caused by alkalinity through urea metabolism. In addition, compared with tilapia fed a high-protein diet in alkaline water, purine metabolism was markedly upregulated in tilapia fed a low-protein diet in alkaline water in this study. One of the reasons may be that a high-protein diet caused a more severe ammonia metabolic burden on tilapia. Carbohydrate metabolism is a vital energy metabolism pathway of fish under unfavorable conditions [53]. Pyruvate metabolism is one of the keys to carbohydrate metabolism [54]. Interestingly, compared with tilapia fed the high-protein diet in alkaline water, pyruvate metabolism was upregulated and protein metabolism was downregulated in tilapia fed the low-protein diet in alkaline water, which meant that tilapia was more inclined to provide energy by carbohydrate metabolism under alkalinity stress. Therefore, the results showed that the low-protein diet with high carbohydrates was more beneficial to ammonia metabolism in tilapia than the high-protein diet with low carbohydrates under alkalinity stress, which may be one of the reasons why tilapia fed the low-protein diet had better growth performance.

## 5. Conclusion

Alkalinity stress will seriously affect the healthy growth of tilapia, and a high-protein diet will increase the burden of ammonia metabolism of tilapia in alkaline water. Reducing dietary protein content by increasing carbohydrates can not only provide energy support for long-term alkalinity culture but also alleviate the ion transport abnormalities and ammonia metabolism abnormalities caused by alkalinity stress. Dietary nutrition strategies of a lower-protein diet with more carbohydrates can effectively improve the growth performance of tilapia under long-term alkalinity stress.

## Figures and Tables

**Table 1:** Formulation and chemical composition of experimental diets (as percent of dry matter).

Ingredients	Diets		
	P27/C35	P35/C25	P42/C15
Fish meal (681 g/kg protein)	80	80	80
Soybean meal (460 g/kg protein)	210	290	370
Corn meal (651 g/kg protein)	180	230	280
Corn oil	44	41	38
Corn starch	350	250	150
Cellulose	54	27	0
Vitamin mix^a^	20	20	20
Mineral mix^b^	20	20	20
Ca(H_2_PO_4_)_2_	10	10	10
Choline chloride	2	2	2
Carboxy methylcellulose	30	30	30
Total	1,000	1,000	1,000
Chemical composition, %			
Moisture	8.96	9.35	11.09
Crude protein	26.91	34.75	41.65
Crude lipid	6.10	5.93	6.08
Ash	6.31	7.09	7.61
Total energy (kJ/g)^c^	14.78	14.84	14.81

^a^Vitamin premix (mg/kg): vitamin A (500,000 IU/g), 8 mg; vitamin D3 (1,000,000 IU/g), 2 mg; vitamin K, 10 mg; vitamin E, 200 mg; thiamine, 10 mg; riboflavin, 12 mg; pyridoxine, 10 mg; calcium pantothenate, 32 mg; nicotinic acid, 80 mg; folic acid, 2 mg; vitamin B12, 0.01 mg; biotin, 0.2 mg; choline chloride, 400 mg; and vitamin C-2-polyphosphate (150 mg/g vitamin C activity), 400 mg. ^b^Mineral premix (mg/kg): zinc (ZnSO_4_·7H_2_O), 50.0 mg; iron (FeSO_4_·7H_2_O), 40 mg; manganese (MnSO_4_·7H_2_O), 15.3 mg; copper (CuCl_2_), 3.8 mg; iodine (KI), 5 mg; cobalt (CoCl_2_·6H_2_O), 0.05 mg; and selenium (Na_2_SeO_3_), 0.09 mg.^c^Calculated values based on 23.6, 39.5, and 17.2 kJ/g for protein, lipid, and nitrogen-free extracts, respectively [25].

**Table 2:** List of the *O. niloticus* primers used for RT-PCR.

Gene	Forward primer (5′–3′)	Reverse primer (5′–3′)	Product size (bp)	GenBank
*β-actin*	AGCCTTCCTTCCTTGGTATGGAAT	TGTTGGCGTACAGGTCCTTACG	102	KJ126772.1
*nka*	CGTGCTGAATTTAAGGCAGGTCA	GCAAAGCTGATTCAGAAGCGTCAC	103	LC556924.1
*vha*	GACCATTCGCTGTCGTAAAC	CCAGCGGTATGTCCTGTTTA	237	AB369668.1
*caIV*	GACAGTGGAAAACAAAGGG	ATTGGGCGGCTCTGTAGT	98	XM_005452652.4
*gs*	CGATCCATTCCGCAAAGA	ACAGGTGAGCCGAAGGTTG	94	AF503208.1
*gls 2*	CTTTTGCTCCAATGACTCTA	CTGCGATGGTGTAGAACAG	104	XM_025902817.1

*nka*, sodium/potassium-transporting ATPase; *vha*, V-type proton pump; *ca IV*, carbonic anhydrase IV; *gs*, glutamine synthetase; and *gls 2*, glutaminase 2.

**Table 3:** Growth performance of *O. niloticus* fed different experimental diets (mean ± SEM, *n* = 3).

Diets	SR (%)	WG (%)	SGR (%)	HSI (%)	CF (g/cm^3^)	FCR
FW- P27/C35	97.33 ± 2.18	6493.58 ± 539.92 ^*∗*^	8.45 ± 0.12^A,B,^ ^*∗*^	1.72 ± 0.04	3.21 ± 0.02^B^	1.27 ± 0.01
FW- P35/C25	86.67 ± 2.18	7207.47 ± 196.53 ^*∗*^	8.68 ± 0.03^A,^ ^*∗*^	1.67 ± 0.05	3.39 ± 0.05^A,B^	1.13 ± 0.02
FW- P42/C15	92.00 ± 3.77	6216.37 ± 222.81 ^*∗*^	8.29 ± 0.07^B,^ ^*∗*^	1.63 ± 0.03	3.50 ± 0.07^A,^ ^*∗*^	1.20 ± 0.05
AW-P27/C35	97.33 ± 1.09	3309.44 ± 7.22^a^	7.00 ± 0.04^a^	1.86 ± 0.05^a^	3.23 ± 0.03^a^	1.70 ± 0.05^c,^ ^*∗*^
AW- P35/C25	93.33 ± 1.09	2402.73 ± 69.50^b^	6.24 ± 0.12^b^	1.80 ± 0.03^a^	3.27 ± 0.03^a^	2.20 ± 0.11^b,^ ^*∗*^
AW- P42/C15	96.00 ± 1.89	905.81 ± 5.05^c^	4.62 ± 0.01^c^	1.44 ± 0.06^b^	3.09 ± 0.01^b^	3.97 ± 0.03^a,^ ^*∗*^
Two-way ANOVA (*P* value)						
Alkalinity	0.136	<0.001	<0.001	0.559	0.002	<0.001
Protein/carbohydrate	0.058	0.002	<0.001	0.001	0.138	<0.001
Interaction	0.489	0.013	<0.001	0.016	0.004	<0.001

Values in the same line with different superscripts letters are significantly different (*P* < 0.05). ^A,B^Indicates a significant difference between dietary protein to carbohydrate ratios within the freshwater (*P* < 0.05). ^a,b,c^Indicates a significant difference between dietary protein to carbohydrate ratios within the alkaline water (*P* < 0.05).  ^*∗*^Indicates a significant difference between alkalinity levels within the same protein–carbohydrate ratios (*P* < 0.05). Survival rate (SR) = 100 × (number of final fish/number of initial fish). WG, weight gain rate = 100 × (final body weight (g) − initial body weight (g))/initial body weight (g). SGR, specific growth rate = 100 × ((ln final body weight – ln initial body weight)/experimental days). HSI, hepatosomatic index = 100 × (wet liver weight (g)/wet body weight (g)). CF, condition factor = 100 × body weight (g)/body length (cm^3^). FCR, feed conversion ratio = feed consumption/(final biomass – initial biomass + dead fish weight).

## Data Availability

Data can be available from the corresponding author upon reasonable request.
